# Supplementary material for: Developmental cues are encoded by the combinatorial phosphorylation of Arabidopsis RETINOBLASTOMA-RELATED protein RBR1
Source: EMBO J. 2024 Oct 28;43(24):6656–78. doi: 10.1038/s44318-024-00282-3 (PMC11649800; doi:10.1038/s44318-024-00282-3)
Supplement: Supplementary file 1 — Appendix [file 44318_2024_282_MOESM1_ESM.pdf]

APPENDIX

Developmental cues are encoded by the combinatorial phosphorylation of *Arabidopsis* RETINOBLASTOMA-RELATED protein RBR1

Jorge Zamora-Zaragoza[1,2], Katinka Klap[1], Jaheli Sánchez-Pérez[3], Jean-Philippe Vielle-Calzada[3], Viola Willemsen[1], Ben Scheres[1,2,*].

[1]Laboratory of Molecular Biology, Department of Plant Sciences, Wageningen University and Research, 6708 PB Wageningen, the Netherlands.
[2]Rijk Zwaan Breeding B.V., Department of Biotechnology, Eerste Kruisweg 9, 4793 RS Fijnaart, the Netherlands.
[3]Laboratorio Nacional de Genómica para la Biodiversidad, Centro de Investigación y de Estudios Avanzados del Instituto Politécnico Nacional, 36824, Irapuato, Guanajuato, Mexico.
*Corresponding author: email: ben.scheres@wur.nl

Table of contents

Appendix Figure S1. RBR protein-protein interactions confirmed by Y2H 2

Appendix Table S1. RBR protein-protein interactions with transcriptional regulators by Y2H 3

Appendix Table S2. Codon changes in RBR amino acid substitutions 4

Appendix Table S3. List of primers 5

Appendix Supplementary Methods 6

References 10

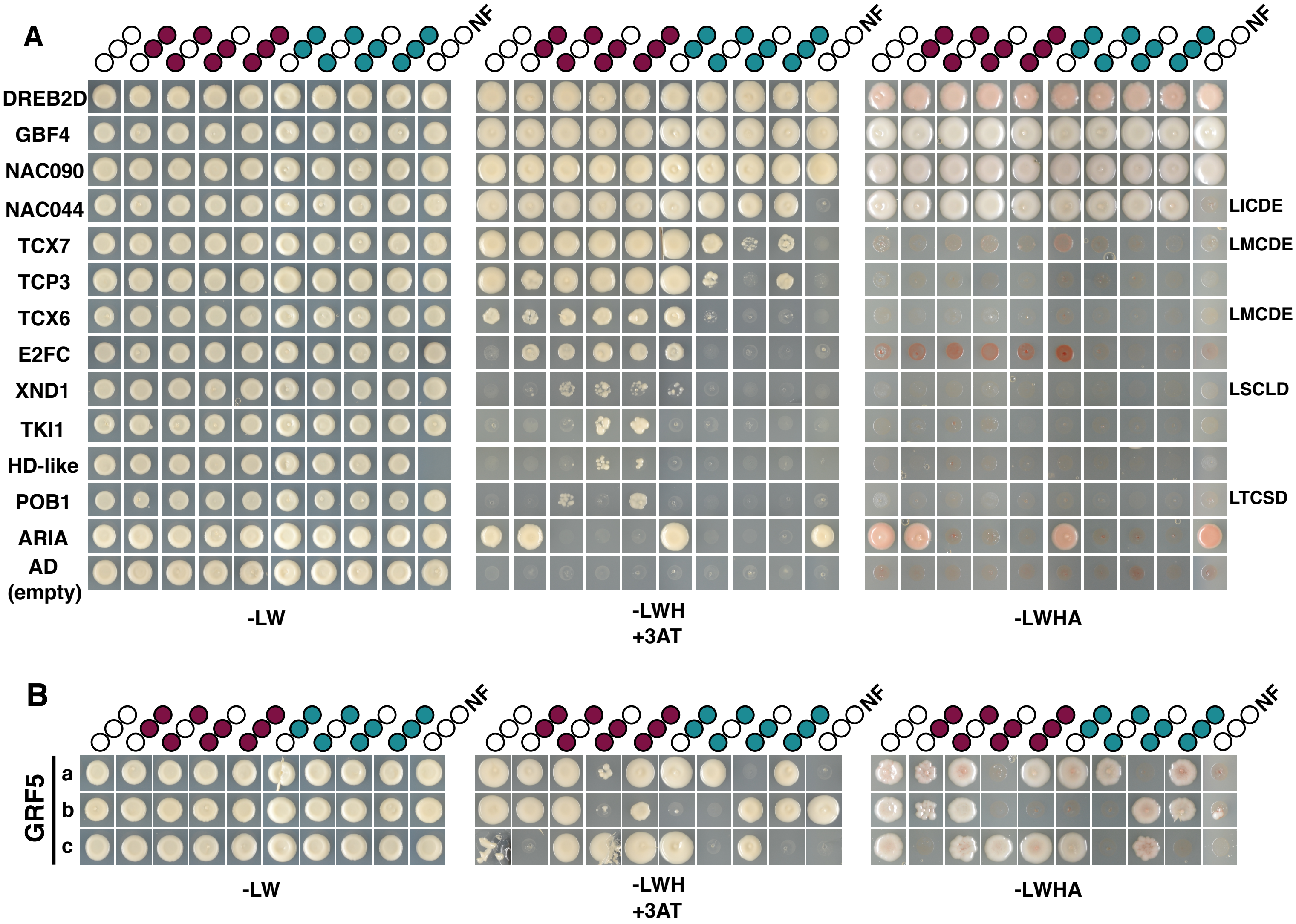

Appendix Figure S1. RBR protein-protein interactions with transcriptional regulators are differentially regulated by phosphorylation as shown by Y2H screenings of the Arabidopsis transcription factors library. A) Y2H analysis of co-transformed pEXP32-RBR variants and pEXP22- TFs found in the initial library screenings, dropped on SD-LW, SD-LWH +1.5mM 3AT and SD –LWHA. The 28 interactors obtained from 10 screenings of the Arabidopsis TF library (Appendix Supplementary methods; and Appendix Table S1) were included in the co-transformation experiment but only those 14 that were confirmed are shown. The identity of each TF fused to the GAL4 activating domain (AD) is indicated on the leftmost column; if present, LXCXE and LXCXE-like motifs sequences are indicated in the rightmost column. Negative control is the empty pDEST22 (AD) vector. B) From the Y2H analysis, the three replicates denoted by lowcase letters show that the GRF5 TF interacted strongly but inconsistently to all RBR variants, possibly reflecting the lack of a co-factor. Co-transformed yeast dropped on SD –LW to select transformants, and on SD -LWH +1.0 mM 3AT and SD -LWHA to select interactions.

Apendix Table S1. List of 28 preys that interacted with RBR baits in Y2H screenings of the Arabidopsis TF library. "x" indicates interaction

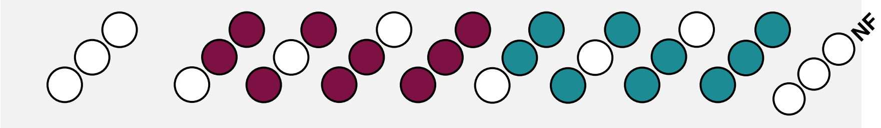

Prays	Gene ID	Baits									
		RBR^{Wt}	Phosho-defective				Phospho-mimetic				RBR^{NF}
**GBF4***	AT1G03970	x									
**E2FC***	AT1G47870		x	x	x	x	x				
**TCP3***	AT1G53230	x		x	x						
**TCP15**	AT1G69690			x		x		x	x	x	
**TCP22**	AT1G72010								x	x	
**DREB2D***	AT1G75490				x						
**HMGB4**	AT2G17560										x
**TCX6***	AT2G20110	x	x	x	x	x	x				
**TKI1***	AT2G36960				x	x					
**NATA2**	AT2G39020			x							
**LBD15**	AT2G40470			x	x	x					
**TLP3**	AT2G47900	x									x
**NAC044***	AT3G01600	x	x	x	x	x	x	x	x	x	
**ATL6**	AT3G05200										x
**GRF5***	AT3G13960					x					
**TCP4**	AT3G15030	x	x	x	x	x	x	x	x	x	x
**TCP14**	AT3G47620		x	x	x	x			x		
**POB1***	AT3G61600					x					
**UNE12**	AT4G02590	x									
**HD-like***	AT4G03250				x						
**OFP9**	AT4G04030					x					
**ERF015**	AT4G31060						x				
**COP3**	AT4G37580				x						
**ZFHD3**	AT5G15210		x								
**ARIA***	AT5G19330	x	x				x				x
**NAC090***	AT5G22380										
**TCX7***	AT5G25790	x	x	x	x	x	x	x			
**XND1***	AT5G64530			x	x	x					
**Total interactions per bait:**		10	8	11	13	13	7	4	5	4	5

*Confirmed interactors (See Appendix Figure S1).

Appendix Table S2. Codon changes in RBR amino acid substitutions.

				Codon change		
**Module**	**AA position**	**Sequence**	**Wt codon**	**Phospho-defective (Ala)**	**Phospho-mimetic (Asp or Glu)**	**Phe**
**N**	T9	PPV**T**PPI	ACC	GCT	GAC	
	S290	KKP**S**PAS	TCT	GCT	GAT	
	S375	ALS**S**PAR	TCA	GCT	GAT	
	S382	TFI**S**PLS	AGC	GCA	GAA	
	S385	SPL**S**PHK	TCT	GCT	GAT	
	S389	PHK**S**PAA	TCG	GCA	GAC	
	T406	LAA**T**PVS	ACA	GCT	GAG	
**P**	S423	TVI**S**PLL	TCC	GCC	GAC	
	S430	PKP**S**PGL	TCT	GCT	GAT	
	S665	GIR**S**PKR	TCG	GCG	GAG	
	S685	SFT**S**PVK	TCA	GCA	GAA	
	S712	AFA**S**PTR	AGC	GCC	GAC	
**C**	S885	CPG**S**PKV	TCG	GCG	GAG	
	S898	PDM**S**PKK	TCC	GCC	GAC	
	S911	VYV**S**PLR	TCT	GCT	GAT	
	S942	AYQ**S**PSK	AGC	GCC	GAC	
	N849	FY**N**EI	AAT	-	-	TTT

Appendix Table S3. List of primers

Primer	Sequence
RBR_n1_WT_F	AAAGGTCTCAACATAATGGAAGAAGTTCAGCCTCCAGT
RBR_n3_WT_R	AAAGGTCTCAACAACTGGTGTTGCTGCCAACTTGGTA
RBR_AB_F	AAAGGTCTCAACATCCAGTGAGCACAGCAATGACAAC
RBR_AB_R	TTTGGTCTCTACAATCTGCACAAGTTTCTCCTCCACCTCC
RBR_C_F	CCCGGTCTCGACATCAGAAACTGGAATCAATATTTTCTT
RBR_C_R	TTTGGTCTCTACAACGAATCTGTTGGCTCGGTTTTAAGGG
RBR_n1_T9D F	AAAGGTCTCAACATAATGGAAGAAGTTCAGCCTCCAGTGGACCCGCCCATTGAACCAAATGGGAAA
RBR_n1_T9A_F	AAAGGTCTCAACATAATGGAAGAAGTTCAGCCTCCAGTGGCTCCGCCCATTGAACCAAATGGGAAA
RBR_n1_S290D_R	CCCGGTCTCGATCTGGCTTTTTCTTCAGTATGGTTTCTA
RBR_n1_S290A_R	GCGGGTCTCGAGCTGGCTTTTTCTTCAGTATGGTTTCTA
RBR_n2_S290D_F	AAAGGTCTCAAGATCCAGCATCTGAGTGCCAAACTGACAAGCTA
RBR_n2_S290A_F	AAAGGTCTCAAGCTCCAGCATCTGAGTGCCAAACTGACAAGCTA
RBR_n2_R	CCCGGTCTCCGCTCAAAGCATCAATTTTCCTCTTA
n3T406A_R	taaGGTCTCtACAACTGGagctgctgccaacttggtagcac
n3T406E_R	taaGGTCTCtACAACTGGctctgctgccaacttggtagcac
GGpRBR_F	aaGAAGACaaGGAGtgcctcgtgtcggaaatatctaattctctctctggatccactcacactcgaagatgacgaagtagacttaatctgaatcc atc
GGpRBR_R	ccgaagacggcattagtctccaacgcagctgaaaacatgcaaaatcaagctaattttacttccaattaaactgctaactgtagacgaagaaaaag ggacttttcaa
GGvYFP_F	ttGAAGACaaTTCGtctgtgagcaagggcgaggagctgttc
GGvYFP_R	gcGAAGACttAAGCttacttgtacagctcgtccat
N849F_F	GAGGTCTCCactttgaaatatttattcctgccgtaaagccg
N849F_R	GGGGTCTCCaagtagaatgtgatgatgtcaacatgatctg
n3RvComp	TGGGGTCTCTACAACTGG
TCX6c-F	GGGGACAAGTTTGTACAAAAAAGCAGGCTATATGGGAGAAGGTGAAGAAGG
TCX6gcg1-F	CAAACGGTCTTTGTTCTGGGAACTGCAAATGCTTGGATTG
TCX6gcg1-R	CAGTTCCCAGAACAAAGACCGTTTGCTTGAAAGCACTCACA
TCX6gcg2-F	TGGCAGGGATGTGTGACGGACGGGACACAATGTTAATGGTT
TCX6gcg2-R	TCCCGTCCGTCACACATCCCTGCCAAAGTTTCTGGAGATAGTG
TCX6c-R	GGGGACCACTTTGTACAAGAAAGCTGGGTTTTAGAGGTCTTTCTTCTCAGACA
cRBR-GWF	GGGGACAAGTTTGTACAAAAAAGCAGGCTTAATGGAAGAAGTTCAGCCTCCAGT
cRBR-GWR	GGGGACCACTTTGTACAAGAAAGCTGGGTAcgaaTCTGTTGGCTCGGTTTTAAG
**n3 synthetic probes**	
RBR_n3-5E	TTTGGTCTCTGAGCGATCCTGCAAGGACATTTATAGAACCACTTGATCCTCATAAGGACCCTGCTGC TAAGACAAATGGTATTAGCGGTGCTACCAAGTTGGCAGCAGAGCCAGTTGTAGAGACCCCA
RBR_n3-5A	TTTGGTCTCTGAGCGCTCCTGCAAGGACATTTATAGCACCACTTGCTCCTCATAAGGCACCTGCTGC TAAGACAAATGGTATTAGCGGTGCTACCAAGTTGGCAGCAGCTCCAGTTGTAGAGACCCCA

Appendix Supplementary Methods

Cloning of RBR phospho-variants

All primers used for cloning, carrying relevant restriction sites and 4 bp overhangs were designed using the Primer3 software (Untergasser et al., 2012) and are listed in Appendix Table S3. The CDS of Wt RBR was amplified in three fragments, namely N0, AB0, C0, with the primer pairs RBR_n1_WT_F / RBR_n3_WT_R, RBR_AB_F / RBR_AB_R, RBR_C_F / RBR_C_R. Each fragment was cloned in level -1 vector pAGM1311 (generating pAGM1311-RBR_N0, pAGM1311-RBR_AB0, and pAGM1311-RBR_C0). To clone the phospho-defective (N-) and phospho-mimetic (N+) mutant modules, we divided the N fragment it in three sub-fragments (namely n1, n2, n3); fragments n1 and n2 were amplified with primers that introduced the corresponding mutation in the phospho-sites T9 and S290: RBR_n1_T9D F / RBR_n1_S290D_R, and RBR_n1_T9A_F / RBR_n1_S290A_R for n1; RBR_n2_S290D_F / RBR_n2_R, and RBR_n2_S290A_F / RBR_n2_R for n2. Mutations in the remaining phospho-sites of module N, were introduced by a synthetic probe, corresponding to n3 fragment, carrying the corresponding mutant codons. The single stranded n3 probes were complemented to double stranded DNA using the n3RvComp primer in a Klenow fragment reaction (Thermo Scientific™, EP0421). Phospho-defective and phospho-mimetic fragments n1, n2, n3 were assembled and cloned into pAGM1311 vector to generate N- and N+ modules (pAGM1311-RBR_N- and pAGM1311-RBR_N+). Phosphorylation mutations in the AB and C regions were obtained amplifying the relevant fragments from pre-existing unpublished phosphorylation mutants (generated by serial rounds of directed mutagenesis) with the same primer pairs as for the wild type fragments. The amplicons were cloned into pAGM1311 (pAGM1311-RBR_AB+, pAGM1311-RBR_AB-, pAGM1311-RBR_C+, pAGM1311-RBR_C-). The NT406 mutant modules were obtained by amplifying the corresponding wild type RBR CDS fragment with the primer pairs RBR_n1_WT_F / n3T406A_R, and RBR_n1_WT_F / n3T406E_R, and cloning into pAGM1311 (pAGM1311-RBR_NT406- and pAGM1311-RBR_NT406+). The N849F mutation was introduced in the C0 and C- modules by amplifying two overlapping fragments from the corresponding level -1 modules with the primer pairs RBR_C_F / N849F_R for the first fragment, and N849F_F / RBR_C_R for the second one; both fragments for each C module (wild type and phospho-defective) were assembled and cloned in pAGM1311 vector (pAGM1311-RBRNF_C0 and pAGM1311-RBRNF_C-). All codon changes mentioned above are listed in Appendix Table S2.

The combinations of level -1 modules specified in Fig S1 were assembled into level 0 vector pAGM1287, creating full length RBR CDS of the corresponding phospho-variant (pAGM1287-RBR_N*AB*C*, where "*" indicates the diverse phospho-modules). For RBR promoter, the intergenic region comprising 1150 bp upstream of the ATG was amplified with primer pair GGpRBR_F / GGpRBR_R, that removed internal BpiI sites, and then cloned into level 0 pICH41295 vector (pICH41294-pRBR). The CDS of the SCFP3A fluorescent protein was amplified with the primer pair GGvYFP_F / GGvYFP_R from an existing clone and sub-cloned into level 0 pAGM1301 vector (pAGM1301-SCFP3A). Each pAGM1287-RBR_N*AB*C* was then combined with pICH41295-pRBR, pAGM1301-SCFP3A, and pICH41421-NosT (from the MoClo toolbox) into level 1 pICH47742 vector (pICH47742-pRBR_RBR_N*AB*C*_SCFP3A_NosT). Level 1 phospho-variants were cloned into level 2 pAGM4723 vector together with the pICH47732-

FAST-R selection marker cassette. All digestion-ligation reactions were performed using 30 fmol of the relevant fragments, plasmids and vector, 1x Green buffer (Thermo Scientific™, No.), 1 μM ATP (Thermo Scientific™), either 1unit/μL BsaI or BpiI enzymes (Thermo Scientific™), T4 DNA ligase (Thermo Scientific™) and water to a final volume of 15 μL.

Yeast two-hybrid (Y2H) analysis: Plasmid construction, transformation, screenings and confirmation of interactions.

We set out to detect RBR protein partners by probing the Y2H library of Arabidopsis transcription factors comprising 1956 nuclear proteins arrayed in 96-well plates (Pruneda-Paz et al., 2014). In total, we used 10 RBR variants as baits: RBR^{Wt}, 4 phospho-defective variants, their 4 phospho-mimetic counterparts, and RBR^{NF}(Fig S5). pEXP32-RBR and pEXP32-RBR^{NF} were reported previously (Cruz-Ramírez et al., 2013). The CDS of the eight phospho-variants was amplified from the corresponding level 0 constructs pAGM1287-RBR_N*AB*C* ("*" indicates the diverse phospho-modules listed in Fig EV1) with the primer pair cRBR-GWF/ cRBR-GWR (Appendix Table S3) and cloned into pDONR221 vector with the Gateway BP clonase II enzyme mix (Invitrogen, 11789020), and the resulting pDONR221-RBR_N*AB*C* phospho-variants entry clones were recombined into pDEST32 by Gateway LR clonase II enzyme mix (Invitrogen, 11791020), resulting in the bait plasmids (pEXP32-RBR_N*AB*C* phospho-variants).

All pEXP32-RBR variants were transformed into yeast strain PJ69-4α and tested for autoactivation as described in (De Folter & Immink, 2011) for at least ten independent transformants; most colonies showed no autoactivation even in selective medium without 3-AT. One colony from each bait with no autoactivation in selective medium supplemented with 0mM 3-AT was inoculated in liquid -L SD-glucose medium and grown O/N. 1mL of the pre-culture was inoculated in 50mL -L SD-glucose medium and grown O/N. In parallel to bait pre-culture, 5 μL of the arrayed Arabidopsis pEXP22-TF library (Pruneda-Paz et al., 2014) was spotted on -W SD-glucose agar plates from PJ69-4A glycerol stock, and grown for 2 days.

A multichannel pipet was used as replicator to transfer the spotted pEXP22-TF library to 96-well plates containing 50 μL of sterile mQ water. 5 μL of the resuspended yeast was spotted on YPD agar plates, letting spots to dry before spotting 5 μL of the pEXP32-RBR variant bait on top and incubating O/N for mating. The grown yeast was then transferred from YPD to 96-well plates containing 50 μL of sterile mQ water, resuspended, and 5 μL spotted on -LW SD-glucose agar plates and incubated for 3 days to select for the presence of both bait and pray plasmids. The transfer procedure was repeated from the -LW SD-glucose to fresh -LW SD-glucose and –LWH + 1.0 mM 3-AT SD-glucose agar plates, and incubated for 4-5 days. Selection of positive interaction was based on at least 3 colonies per spot. From the 10 independent library screenings, we identified 28 interactors in total (Appendix Table S1). To confirm the identity of the positive interactors, plasmid was extracted from the yeast colonies, transformed in chemically competent *E. coli* DH5-α, selected in LB + ampicillin and mini-prepped again for sequencing.

To assess the interaction patterns, the bait plasmids (pEXP32-RBR variants) were transformed into yeast strain PJ69-4A as described in (De Folter & Immink, 2011). Not autoactivating colonies in 0mM 3-AT, carrying each of the baits, were made competent again and transformed with each of the 28 purified and sequenced pEXP22-TF preys obtained from the screenings. The transformation was adapted from (De Folter & Immink, 2011) to be done in 96 deep well plates instead of 1.5 mL tubes. Transformed yeast was resuspended in 150 μL of sterile mQ water and spotted in triplicate on -LW SD-glucose agar plates and incubated for 3 days. The yeast was then transferred to 96-well plates containing 50 μL of sterile mQ water, resuspended, and 5 μL spotted onto fresh -LW SD-glucose, –LWH + 1.5 mM 3-AT SD-glucose, and -LWHA SD-glucose agar plates, and incubated for 5 days. Selection of positive interaction was based on at least 3 colonies per spot. 14 of the initial 28 interactors consistently failed to show interaction with any of the RBR variants in the confirmation by co-transformation experiment.

For pEXP22-TCX6^{gcg1} and pEXP22-TCX6^{gcg2}, we first generated the corresponding entry clones by amplifying the attB-flanked TCX6 CDS in a two-fragments overlapping PCR to mutagenize the LXCXE and LXCXE-like motifs with the primer pairs TCX6c-F/ TCX6gcg1-R and TCX6gcg1-F /TCX6c-R for TCX6^{gcg1}; and TCX6c-F/TCX6gcg2-R and TCX6gcg2-F /TCX6c-R for TCX6^{gcg2} (Appendix Table S3) followed by BP-II clonase (Invitrogen) recombination reaction into pDONR-221. Entry clones were recombined into pDEST22 destination vector with the LR-II clonase (Invitrogen). Small scale Y2H assays was performed by co-transforming bait and pray plasmids as described in (De Folter & Immink, 2011), testing 12 independent co-transformations per interaction. All yeast incubations described above were at 30 °C. Plates were imaged at with a table top flatbed scanner (EPSON Expression 11,000 XL).

Western blot analysis

Total proteins from the genotypes indicated in Fig EV2A were extracted from 7 das seedlings germinated and grown as described above and using the Pierce Plant Total Protein Extraction Kit (Thermo Fisher Scientific, A44056) following the manufacturer's instructions slightly modified. Briefly, Seedlings were flash-frozen and kept at -80 °C until the extraction day, on which they were ground using mortar and pestle before adding the denaturing lysis buffer freshly supplemented with Halt™ Protease and Phosphatase Inhibitor Cocktail (Thermo Fisher Scientific, 78440) and MG-132 (Merck Life Science N.V., M7449), after which we followed the manual procedure. Equal amounts of proteins, as determined with the Pierce™ Rapid Gold BCA Protein Assay Kit (Thermo Fisher Scientific, A53227) were loaded to SDS-PAGE gel 10% (Bio-Rad, Cat. #4568033) and proteins were transferred onto polyvinylidene difluoride membranes (Bio-Rad, Cat #1620174) for 16 h at 30 V in a wet transfer tank (Bio-Rad). The membranes were incubated with the corresponding antibodies diluted in iBind™ Flex Solution Kit (Thermo Fisher Scientific, SLF2020X4) using the iBind™ Flex Western Device (Thermo Fisher Scientific, SLF2000) and iBind™ Flex Cards (Thermo Fisher Scientific, SLF2010X4). The primary anti-GFP Polyclonal antibody (Thermo Fisher Scientific, A-6455) was used in a 1:1000 dilution, and the HRP-coupled goat anti-rabbit secondary antibody (Thermo Fisher Scientific, #31466) in a 1:5000 dilution. After antibodies incubation, the membrane was washed in mQ water fon 1 minute and the SuperSignal West Pico Plus chemiluminescence substrate (Thermo Fisher Scientific, 34580) was applied according to the

manufacturer’s instructions before imaged with the iBright™ CL750 Imaging System (Thermo Fisher Scientific, A44116).

References

Cruz-Ramírez, A., Diaz-trivino, S., Wachsman, G., Du, Y., Arteaga-Vazquez, M., Zhang, H., Benjamins, R., Blilou, I., Neef, A. B., Chandler, V., & Scheres, B. (2013). A SCARECROW-RETINOBLASTOMA Protein Network Controls Protective Quiescence in the Arabidopsis Root Stem Cell Organizer. *PLoS Biology*, *11*(11), e1001724. https://doi.org/10.1371/journal.pbio.1001724

De Folter, S., & Immink, R. G. H. (2011). Yeast protein-protein interaction assays and screens. *Methods in Molecular Biology*, *754*(June), 145–165. https://doi.org/10.1007/978-1-61779-154-3_8

Pruneda-Paz, J. L. L., Breton, G., Nagel, D. H. H., Kang, S. E. E., Bonaldi, K., Doherty, C. J. J., Ravelo, S., Galli, M., Ecker, J. R. R., & Kay, S. A. A. (2014). A Genome-Scale Resource for the Functional Characterization of Arabidopsis Transcription Factors. *Cell Reports*, *8*(2), 622–632. https://doi.org/10.1016/j.celrep.2014.06.033

Untergasser, A., Cutcutache, I., Koressaar, T., Ye, J., Faircloth, B. C., Remm, M., & Rozen, S. G. (2012). Primer3-new capabilities and interfaces. *Nucleic Acids Research*, *40*(15), 1–12. https://doi.org/10.1093/nar/gks596
