# Supplementary material for: Developmental cues are encoded by the combinatorial phosphorylation of Arabidopsis RETINOBLASTOMA-RELATED protein RBR1
Source: EMBO J. 2024 Oct 28;43(24):6656–78. doi: 10.1038/s44318-024-00282-3 (PMC11649800; doi:10.1038/s44318-024-00282-3)
Supplement: Supplementary file 5 — Expanded View Figures [file 44318_2024_282_MOESM5_ESM.pdf]

Expanded View Figures

Figure EV1. Modules and RBR-phosphovariants.

(**A**) Schematic list of all modules cloned in Level -1 (vector pAGM1311) of the GoldenGate MoClo system. Text nomenclature and colored circles and their relative position within the gray background box indicate the phosphorylation state and position of each module within the full-length CDS of phosphovariants according to Fig. 1. Note that in modules "406" only the Thr406 residue is mutated, and in modules "^{NF}C" the Asn849 within the LXCXE-binding cleft of the B-pocket sub-domain is mutated to Phe. (**B**) Schematic list of all phosphovariants generated. All variants listed exist as Level 0 (vector pAGM1287), Level 1 (vector pICH47742; with RBR promotor, SCFP3A CDS and NOS terminator), and Level 2 (vector pAGM4723; with FAST-R selection cassette in position 1) constructs, and as transgenic seed, except for those marked with the symbol ◎ on the left-most column, which were either not viable or not transformed and thus, only the plasmids are available. Note that [406-,P0,C-]^{5} is marked with double circle because reduced fertility hindered propagation and all seed was used for the experiments reported in Fig. 5.

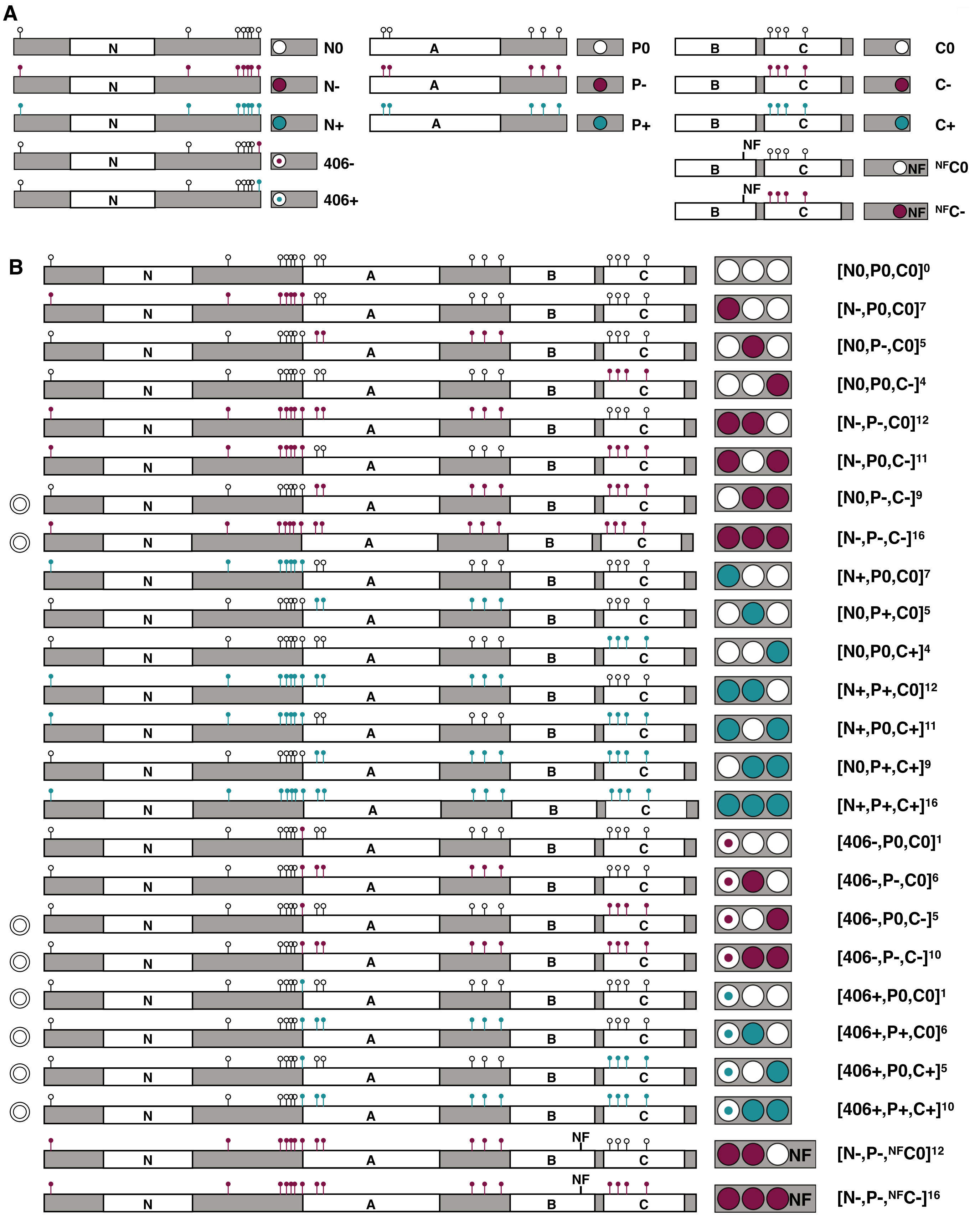
A
N
N0
A
P0
B
C
C0
N-
P-
C-
N+
P+
C+
406-
NF
NFC0
406+
NFC-
B
[N0,P0,C0]0
[N-,P0,C0]7
[N0,P-,C0]5
[N0,P0,C-]4
[N-,P-,C0]12
[N-,P0,C-]11
[N0,P-,C-]9
[N-,P-,C-]16
[N+,P0,C0]7
[N0,P+,C0]5
[N0,P0,C+]4
[N+,P+,C0]12
[N+,P0,C+]11
[N0,P+,C+]9
[N+,P+,C+]16
[406-,P0,C0]1
[406-,P-,C0]6
[406-,P0,C-]5
[406-,P-,C-]10
[406+,P0,C0]1
[406+,P+,C0]6
[406+,P0,C+]5
[406+,P+,C+]10
[N-,P-,NFC0]12
[N-,P-,NFC-]16

 © The Author(s)

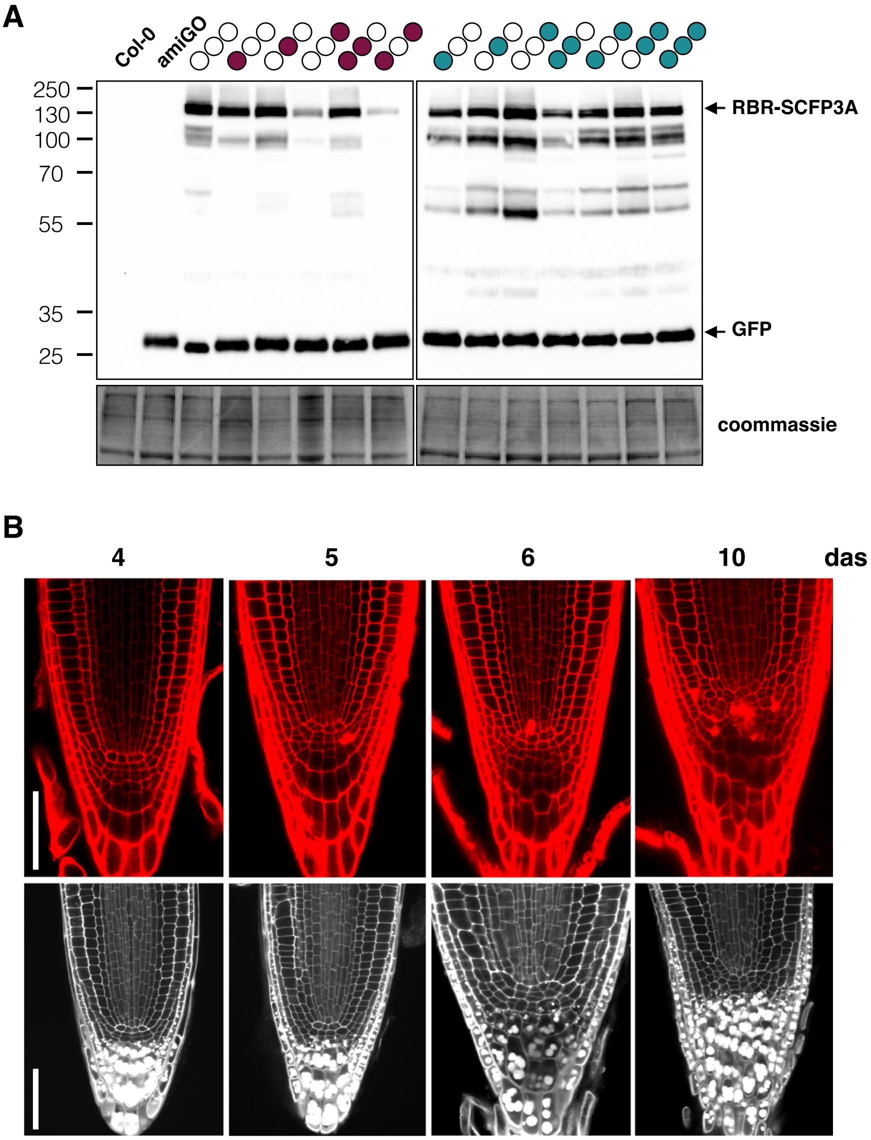

Figure EV2. Characterization of amiGO-RBR phenotype penetrance and RBR phosphovariants protein accumulation.

(**A**) Western blot analysis of RBR phosphorylation variants. The anti-GFP antibody was used to detect the SCFP3A-tagged RBR transgenic variants and the free GFP co-expressed in all lines containing the amiGO construct. (**B**) Confocal images of amiGO-RBR root tips by 4, 5, 6 and 10 days after sowing (das). Top panels, mPS-PI staining; bottom panels, PI staining. Red spots in the SCN area correspond to cell death, as PI selectively stains dead cells. Note that by 5 das we detected roots with very weak or no amiGO-associated phenotypes. By 6das, cell death and SCN extra divisions were evident in all roots. Scale bars, 50 µm.

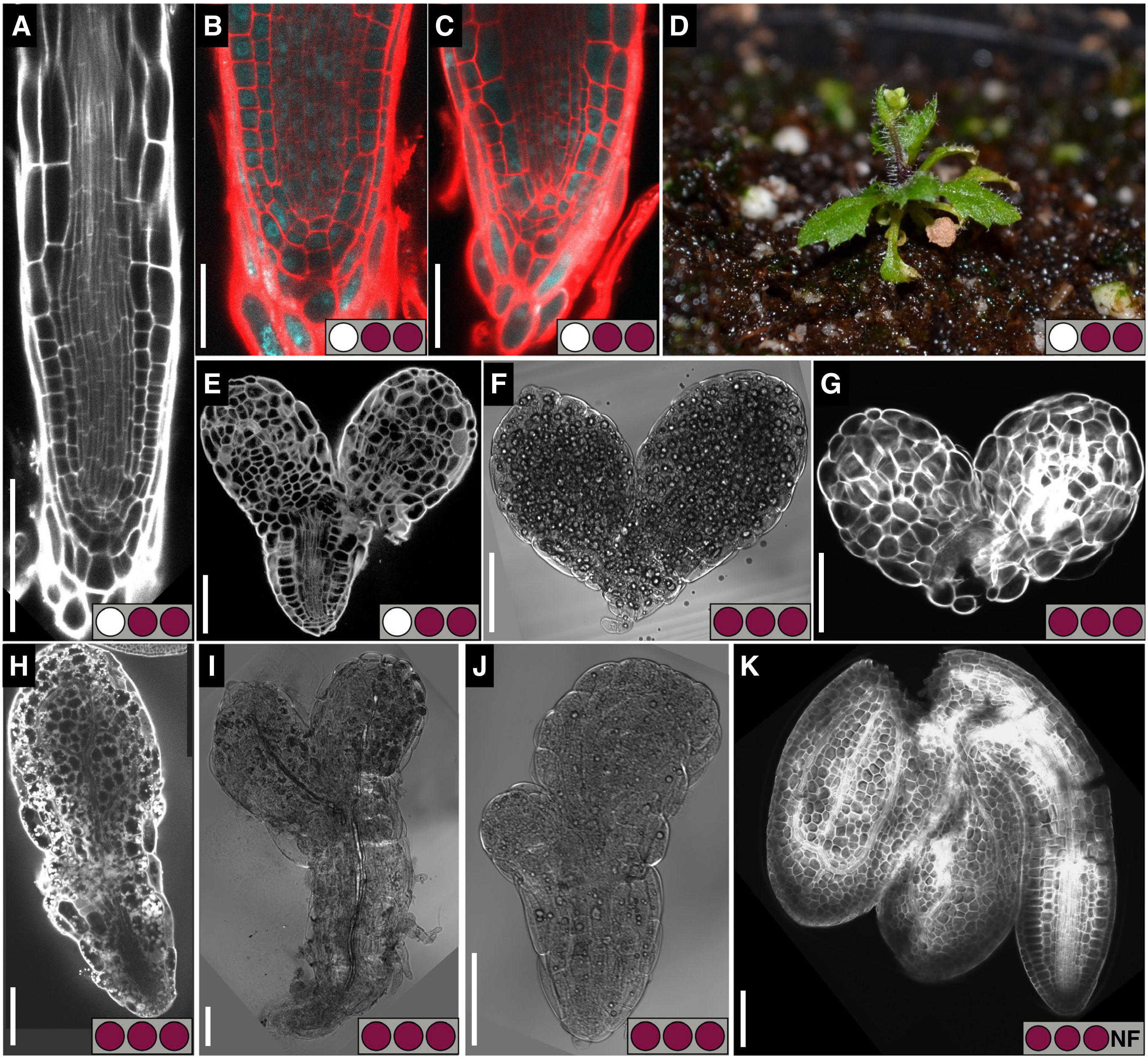

Figure EV3. Primary transformants of lethal phospho-defective RBR variants.

(**A–C**) Confocal images of PI-stained root tips. Max power and gain for CLSM settings and image brightness and contrast in (**B**, **C**) were set to in order to visualize SCFP3A signal from [N0,P-,C-][9] expression (Cyan). (**D**) 3 week old seedling. (**E–K**) Confocal images of mPS-PI-stained (**E**, **G**, H, **K**), and transmitted light images (**F**, **I**, **J**) of embryos from non-germinated seeds 4 das stratified for 4 days. Genotypes: [N0,P-,C-][9] (**A–E**), [N-,P-,C-][16] (**F–J**), [N-,P-^{NF},C-][16] (**K**); Genetic background: Col-0 (**A**, **B**, **G**, **K**), amiGO (**C–F**, **H–J**). Scale bars, 100 μm in (**A**, **E–K**), 50 μm in (**B**, **C**).

 © The Author(s)

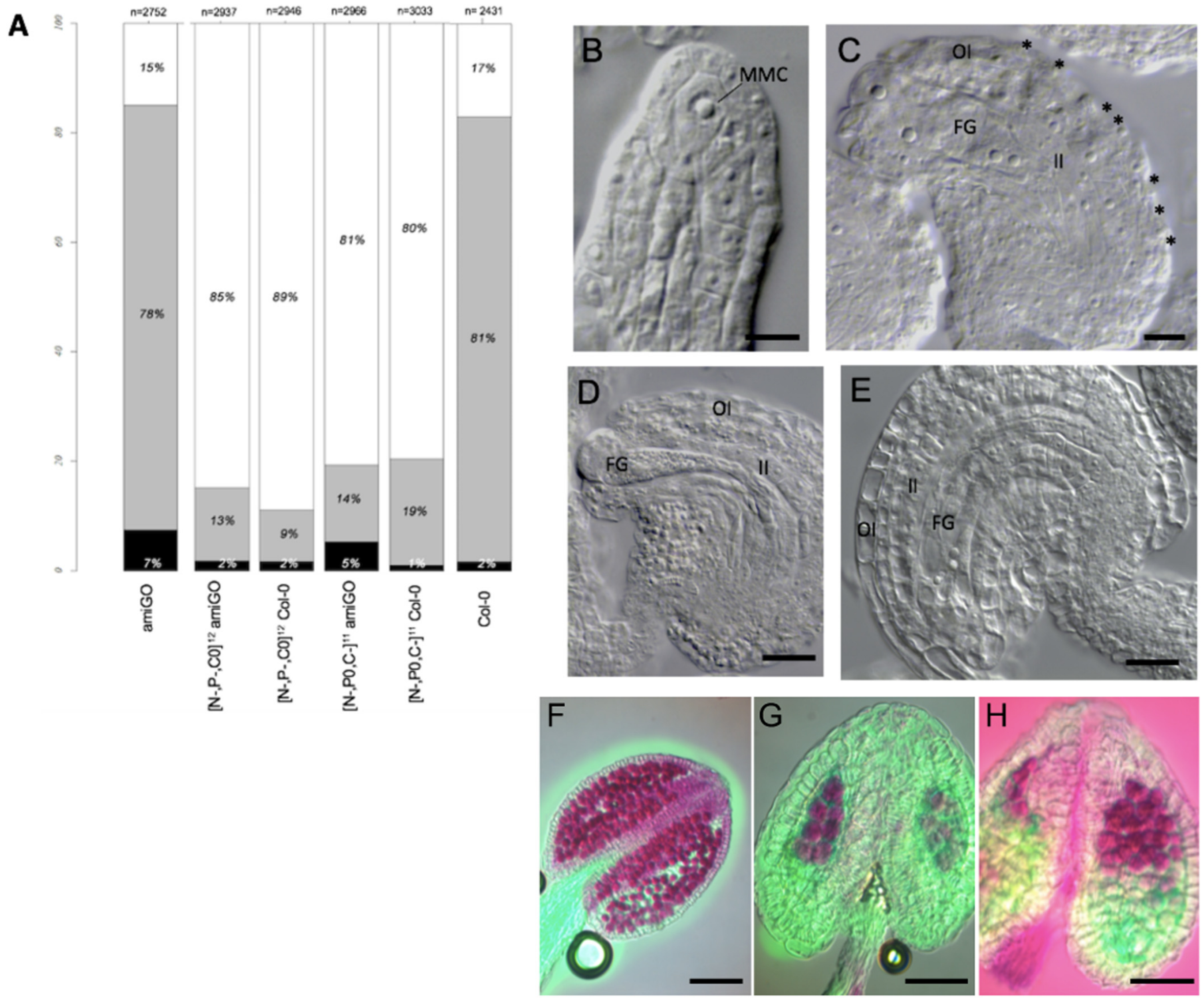

Figure EV4. Fertility and embryogenesis are compromised in the highly substituted RBR phospho-defective variants [N-,P-,C0]^{12} and [N-,P0,C-]^{11}.

(**A**) Sterility analysis quantified as percentage of non-fertilized ovules, aborted seed and mature seed. N denotes total number of scored ovules and seed. (**B–E**) DIC images of ovule development of [N-,P0,C-]^{11} (**B**, **E**) and [N-,P-,C0]^{12} (**C**, **D**). Ovule primordia with one normal precursor cell (**B**). Incomplete integument development results in abnormally exposed embryo sac (**C–E**). Scale bars 10 µm in (**B**), 20 µm in (**C–E**). (**F–H**) Alexander staining of Col-0 (**F**), [N-,P-,C0]^{12} (**G**), and [N-,P0,C-]^{11} (**H**) anthers showing viable pollen grains in fuchsia. Scale bars 100 µm.

© The Author(s)

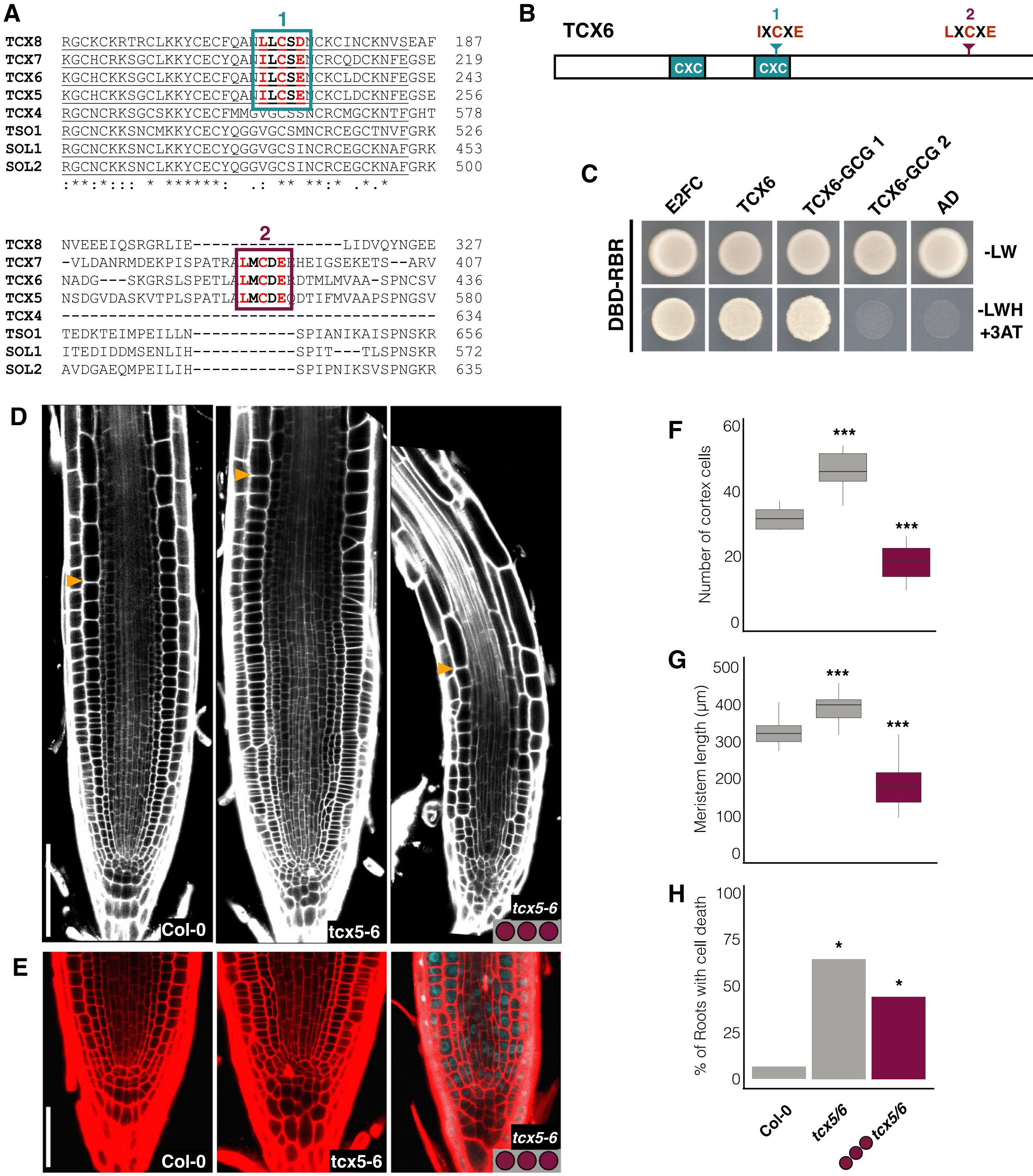
A
1
TCX8 RGCKCKRTRCLKKYCECFQANLLCSDNCKCINCKNVSEAF 187
TCX7 KGCHCRKSGCLKKYCECYQANILCSENCRCQDCKNFEGSE 219
TCX6 KGCHCKKSGCLKKYCECFQANILCSENCKCLDCKNFEGSE 243
TCX5 KGCHCKKSGCLKKYCECFQANILCSENCKCLDCKNFEGSE 256
TCX4 RGCNCRKSGCSKKYCECFMMGVGCSSNCRCMGCKNTFGHT 578
TSO1 RGCNCKKSNCMKKYCECYQGGVGCSMNCRCEGCTNVFGRK 526
SOL1 RGCNCKKSNCLKKYCECYQGGVGCSINCRCEGCKNAFGRK 453
SOL2 RGCNCKKSNCLKKYCECYQGGVGCSINCRCEGCKNAFGRK 500
2
TCX8 NVEEEIQSRGRLIE---------------LIDVQYNGEE 327
TCX7 -VLDANRMDEKPISPATRALMCDEPHEIGSEKETS--ARV 407
TCX6 NADG---SKGRSLSPETLALMCDERDTMLMVAA-SPNCSV 436
TCX5 NSDGVDASKVTPLSPATLALMCDEQDTIFMVAAPSPNGSV 580
TCX4 -- 634
TSO1 TEDKTEIMPEILLN-----------SPIANIKAISPNSKR 656
SOL1 ITEDIDDMSENLIH-----------SPIT---TLSPNSKR 572
SOL2 AVDGAEQMPEILIH-----------SPIPNIKSVSPNGKR 635
B
TCX6
1
IXCXE
2
LXCXE
CXC
CXC
C
E2FC
TCX6
TCX6-GCG 1
TCX6-GCG 2
AD
DBD-RBR
-LW
-LWH
+3AT
D
Col-0
tcx5-6
tcx5-6
E
Col-0
tcx5-6
tcx5-6
F
Number of cortex cells
60
40
20
0
***
***
G
Meristem length (µm)
500
400
300
200
100
0
***
***
H
% of Roots with cell death
100
75
50
25
0
*
*
Col-0
tcx5/6
tcx5/6

 © The Author(s)

◀ **Figure EV5. Phosphorylation-regulated functions of RBR are largely mediated by the interaction with members of the DREAM complex.**

(**A**) Clustal omega multiple sequence alignment fragments of Arabidopsis TCX proteins showing LXCXE-like motifs (red) within the conserved CXC domain (underlined) and LXCXE motifs (in green) within a less conserved region; asterisks and dots indicate identical and similar residues, respectively. (**B**) Schematic representation of TCX6 protein organization as predicted by Pfam server (https://pfam.xfam.org/), showing the relative positions of the cysteine-rich domains (cxc) and LXCXE and LXCXE-like motifs. (**C**). Yeast two-hybrid analysis showing that RBR interacts with TCX6 and a TCX mutated on the LXCXE-like motif '1' within the CXC domain, but not with TCX6 mutated on the canonical LXCXE motif '2'. E2FC is positive control, and empty pDEST22 vector is negative control. Co-transformed yeast dropped on SD –LW to select transformants, and on SD -LWH + 1.0 mM 3AT to select interactions. (**D**, **E**) Representative confocal images of PI-stained root tips of the indicated genotypes; yellow arrowheads mark the end of the meristem proliferation zone. The CLSM settings for detecting SCFP3A in the *tcx5/6*;[N-,P-,C-][16] were identical than those for all other phosphovariants, but brightness and contrast were enhanced to visualize the nuclear signal due to the low fluorescence intensity. Red spots in the SCN area correspond to cell death, as PI selectively stains dead cells. Scale bars, 100 μm in (**D**), 50 μm in (**E**). (**F**, **G**) Box plots from (**D**) of meristem proliferation and size quantified as the number of cortex cells (**F**) and length (**G**) from the QC to the first rapidly elongating cortex cell. (**H**) Bar graph from (**E**) showing the percentage of root tips with dead cells. Data information: Data from one biological replicate presented as median (center line), interquartile range (box) and minina and maxima values (whiskers) in (**F**, **G**); or as means in (**H**); $n > 15$ in (**F**, **G**, **H**). Wilcoxon test against Col-0, $***P < 0.001$ in (**F**, **G**), Chi-square, $*P < 0.05$ in (**H**).

© The Author(s)
